# IGF-1 and hyperglycaemia-induced FOXA1 and IGFBP-2 affect epithelial to mesenchymal transition in prostate epithelial cells

**DOI:** 10.18632/oncotarget.27650

**Published:** 2020-06-30

**Authors:** Rehanna Mansor, Jeff Holly, Rachel Barker, Kalina Biernacka, Hanna Zielinska, Anthony Koupparis, Edward Rowe, Jon Oxley, Alex Sewell, Richard M. Martin, Athene Lane, Lucy Hackshaw-McGeagh, Claire Perks

**Affiliations:** ^1^ IGFs and Metabolic Endocrinology Group, Bristol Medical School, Translational Health Sciences, University of Bristol, Southmead Hospital, Bristol, UK; ^2^ Faculty of Medicine, Royal College of Medicine Perak, Universiti Kuala Lumpur, Ipoh, MY; ^3^ Department of Urology, Bristol Urological Institute, Southmead Hospital, Bristol, UK; ^4^ Department of Cellular Pathology, North Bristol NHS Trust, Southmead Hospital, Bristol, UK; ^5^ NIHR Biomedical Research Centre, Level 3, University Hospitals Bristol Education Centre, Bristol, UK; ^6^ Population Health Sciences, University of Bristol, Bristol, UK

**Keywords:** hyperglycaemia, prostate cancer, FOXA1, IGFBP-2, EMT

## Abstract

Localized prostate cancer (PCa) is a manageable disease but for most men with metastatic disease, it is often fatal. A western diet has been linked with PCa progression and hyperglycaemia has been associated with the risk of lethal and fatal prostate cancer.

Using PCa cell lines, we examined the impact of IGF-I and glucose on markers of epithelial-to-mesenchymal transition (EMT), migration and invasion. We examined the underlying mechanisms using cell lines and tumour tissue samples.

IGF-I had differential effects on the process of EMT: inhibiting in normal and promoting in cancer cells, whereas hyperglycamia alone had a stimulatory effect in both. These effects were independent of IGF and in both cases, hyperglycaemia induced an increase IGFBP-2(tumour promoter) and FOXA1. A positive correlation existed between levels of IGFBP-2 and FOXA1 in benign and cancerous prostate tissue samples and *in vitro* and *in vivo* data indicated that FOXA1 strongly interacted with the *IGFBP-2* gene in normal prostate epithelial cells that was associated with a negative regulation of IGFBP-2, whereas in cancer cells the level of FOXA1 associating with the *IGFBP-2* gene was minimal, suggesting loss of this negative regulation.

IGF-I and hyperglycaemia-induced FOXA1/IGFBP-2 play important roles in EMT.

## INTRODUCTION

Despite advances in the detection and management of prostate cancer, tumour recurrence is common and often progresses to hormone refractory metastatic disease that accounts for most prostate cancer-associated mortalities. Epithelial-to-mesenchymal transition (EMT) appears to play a crucial role in the progression of metastatic prostate cancer [1]. During EMT, epithelial cancer cells lose their cell-cell adhesion and acquire the motile and invasive characteristics of mesenchymal cells that are essential for dissemination and distant metastasis [2]. Loss of epithelial markers such as E-cadherin and β-catenin together with the acquisition of mesenchymal markers such as vimentin and fibronectin are the hallmarks of EMT activation.

The causes of the development of clinically aggressive prostate cancer are not entirely understood, but it was proposed that apart from genetic mutations, adoption of a western lifestyle contributes to the increase in prevalence and progression to clinical disease. Studies have shown an association between metabolic syndrome and an increased risk of prostate cancer progression to advanced disease and prostate cancer-specific mortality [3, 4]. Epidemiologic evidence suggest that suffering from diabetes is associated with a higher risk of developing, and mortality from, many types of cancers including liver, pancreatic, colon, endometrial, kidney and breast [5]. A recent meta-analysis revealed that prostate cancer patients with pre-existing diabetes have a worse prognosis and higher mortality [6]. The underlying mechanisms linking diabetes and cancer development are yet to be fully determined; but it has been suggested that hyperglycaemia, a hallmark of diabetes may promote tumour progression [7].

Growing evidence has shown that dysregulation of the insulin-like growth factor (IGF) signalling pathway contributes to cancer progression and malignant transformation [8]. Epidemiology studies have consistently shown an association between circulating plasma IGF-I with the increased risk and progression of prostate cancer [9–12, 13]. Immunohistochemistry analysis of serial resected prostate cancer specimens found an increase in the IGF receptor (IGF-IR) in androgen-independent metastatic disease [14]. IGFBP-2, the second most abundant IGF-binding protein (IGFBP) in serum after IGFBP-3, plays a crucial role in modulating IGF-activity. IGFBP-2 binding to IGFs regulates the bioavailability of the ligands to the receptors and their half-life in the circulation [15]. Apart from its IGF-dependent actions, IGFBP-2 also exerts effects on cell growth and metabolism independent of IGF [16]. We have reported that IGFBP-2 can promote prostate tumour progression by acting as a growth promoter, a survival factor against docetaxel-induced cell death [17] and that hyperglycaemia-induced up-regulation of IGFBP-2 conferred chemo-resistance in prostate cancer cells [18]. FOXA1 has been identified as a “pioneer factor” that binds to the chromatin-packaged DNA and opens the chromatin for binding of additional transcription factors including the androgen receptor (AR) [19]. Reports on the role of FOXA1 in cancer have been controversial. FOXA1 has been shown to have positive and negative effects on cancer progression [20–24] and is also an important mediator of IGF-I mediated biological responses. In response to IGF-I activation AKT phosphorylates FOXA1 promoting its association with 14-3-3 proteins resulting in its exclusion from the nucleus, thus preventing its actions on DNA. IGF-I regulated genes are enriched with FOXA1 binding sites and FOXA1 suppression inhibits the ability of IGF-I to regulate gene expression involved in cell proliferation and survival via both MAPK and AKT signaling pathways [25]. In this study, we aimed to assess the effects of hyperglycaemia and IGF-I on EMT in normal and malignant prostate epithelial cells. We also investigated the potential mechanism that mediated the effect of hyperglycaemia on EMT by assessing the role of IGFBP-2 and FOXA1.

## RESULTS

### The effect of IGF-I on EMT markers in prostate epithelial cells in altered glucose conditions

We first assessed the differential abundance of epithelial and mesenchymal proteins in a panel of prostate cancer cell lines and in a non-tumourigenic PNT2 prostate epithelial cell line (Supplementary Figure 1A). In agreement with a study by Murali in 2012 [26], E-cadherin was highly abundant in LNCaP cells, with lower levels in the more aggressive VCaP and DU145 cells. The most mesenchymal, PC3 cells had no E-cadherin but did possess high levels of the mesenchymal marker N-cadherin. β-catenin and vimentin were found in all the cell lines, with lower and higher abundance respectively in the more aggressive VCaP, DU145 and PC3 cells. It is also interesting to note, that despite the non-tumourigenic characteristic of PNT2, these cells expressed very low levels of E-cadherin and high levels of N-cadherin suggesting some intrinsic phenotypic plasticity for EMT. From our panel of cell lines, we chose to use PNT2 cells as a representative of normal prostate epithelial cells and DU145 cells which represent an androgen receptor negative prostate cancer cell line to compare if there were any differences in response to hyperglycaemia and IGF-I with these two cell lines.

**Figure 1:**
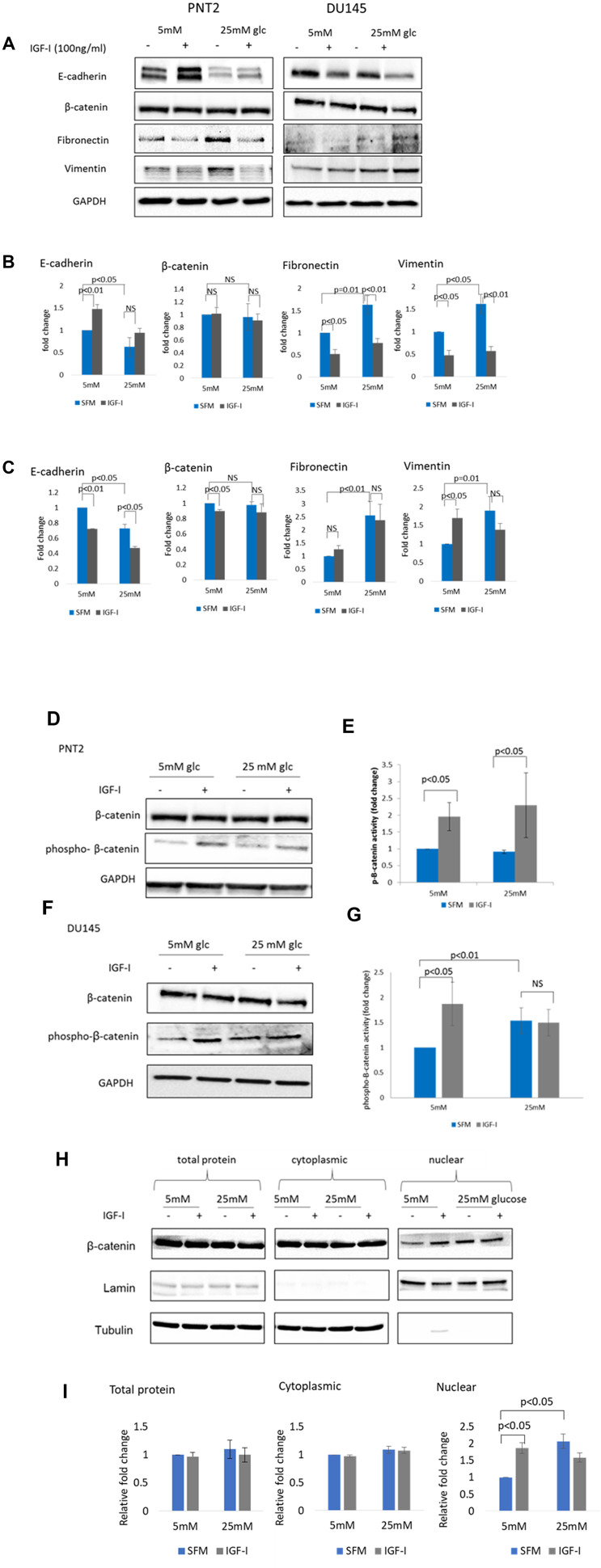
The effect of IGF-I on EMT markers in prostate epithelial cells in altered glucose condition. (**A**) Western blot image shows the effect of IGF-I and high glucose on mesenchymal markers in PNT2 and DU145 cells. Cells were dosed with IGF-I 100 ng/ml for 48 hours in normal (5 mM) and high (25 mM) glucose serum free media. Equal amounts of extracted proteins were separated by SDS-PAGE, blotted to a nitrocellulose membrane. We then cut the membrane into strips, horizontally according to molecular weight markers, to probe the membrane with different antibodies for different sized proteins of interest: E-cadherin, β-catenin, fibronectin, vimentin and GAPDH. GAPDH was used as a loading control. The framed boxes sometimes include areas larger than the strips and therefore appear as no background. Optical densities of protein blots for (**B**) PNT2 and (**C**) DU145 were quantitated using image J and normalised to GAPDH. Western blots showing regulation of p-β-catenin in (**D**) PNT2 and (**E**) DU145 cells when treated with 100 ng/ml IGF-I in normal (5 mM) and high (25 mM) glucose serum free media. Optical densities of protein blots for (**F**) PNT2 and (**G**) DU145 were quantitated using image J and normalised to GAPDH. Ratio of normalised total β-catenin: p- β-catenin were measured and used as an indicator of β-catenin activity. The data expressed as fold changes relative to control represent mean ± SE of triplicate experiments. (**H**) Western blot showing cytosolic and nuclear fractions of protein separated form whole cells lysate (total protein) from DU145 cells treated or untreated with 100 ng/ml IGF-I for 48 hours in normal (5 mM) and high (25 mM) glucose serum free media. Lamin A/C and tubulin act as nuclear and cytoplasmic loading controls respectively. (**I**) Optical densities of protein blots were quantitated using image J and normalised to tubulin/lamin. Results shown are representative of three independent experiments. Data are represented as mean ± SEM.

With PNT2 cells, grown in 5 mM glucose, there was a significant (*p* < 0.01) increase in E-cadherin abundance and a reduction (*p* < 0.05) in fibronectin and vimentin respectively following treatment with IGF-I for 48 hours compared to the untreated controls. No changes in β-catenin levels were observed (Figure 1A and 1B). These findings suggest that IGF-I has an inhibitory effect on EMT and induces mesenchymal-to-epithelial transition (MET) in PNT2 cells.

In contrast, opposite effects were observed with DU145 in 5 mM glucose. IGF-I induced EMT in DU145 cells as shown by a significant reduction of E-cadherin and β-catenin abundance respectively (*p* < 0.01 and *p* < 0.05). The reduction in these epithelial markers was also accompanied by a significant (*p* < 0.05) increase in the mesenchymal marker vimentin but no changes in the level of fibronectin were observed (Figure 1A and 1C).

With PNT2 cells grown in 25 mM glucose, high glucose alone altered some of the EMT markers: it reduced E-cadherin (*p* < 0.05), increased fibronectin and vimentin (*p =* 0.01 and *p* < 0.05 respectively) and had no effect on β-catenin. Despite high glucose alone promoting EMT, IGF-I still decreased both fibronectin and vimentin and had no effect on β-catenin or E-cadherin (Figure 1A and 1C)

With DU145 cells, 25 mM glucose alone promoted EMT with a significant increase in the mesenchymal markers fibronectin and vimentin (*p* < 0.01 and *p =* 0.01) compared to untreated control in normal glucose conditions. E-cadherin levels were also decreased (*p* < 0.05) but there were no significant change in β-catenin levels. Treatment with IGF-I in high glucose conditions resulted in a further decrease in E-cadherin levels (*p* < 0.05) but there were no significant changes observed in fibronectin and vimentin when compared to the untreated high glucose controls (Figure 1A and 1C).

### The effect of hyperglycaemia and IGF-I on β-catenin phosphorylation and subcellular localisation

With PNT2 cells, β-catenin phosphorylation was increased by IGF-I in both normal and high glucose conditions and this was not affected by levels of glucose (Figure 1D and 1E). With DU145 cells, high glucose alone was able to significantly induce β-catenin phosphorylation (*p* < 0.01) when compared to untreated controls in normal glucose conditions. Treatment with IGF-I promoted β-catenin phosphorylation in normal levels of glucose but in high glucose conditions was unable to further increase the phosphorylation when compared to high glucose alone, although the level was still high when compared to untreated control in normal glucose conditions (Figure 1F, 1G). High glucose alone promoted nuclear localisation of β-catenin in DU145 cells (Figure 1H and 1I). Dosing with IGF-I in normal glucose conditions increased translocation of β-catenin to the nucleus whereas in high glucose conditions the addition of IGF-I had no further effect.

### The effect of IGF on cell proliferation and migration in prostate epithelial cells in altered glucose condition

**Figure 2:**
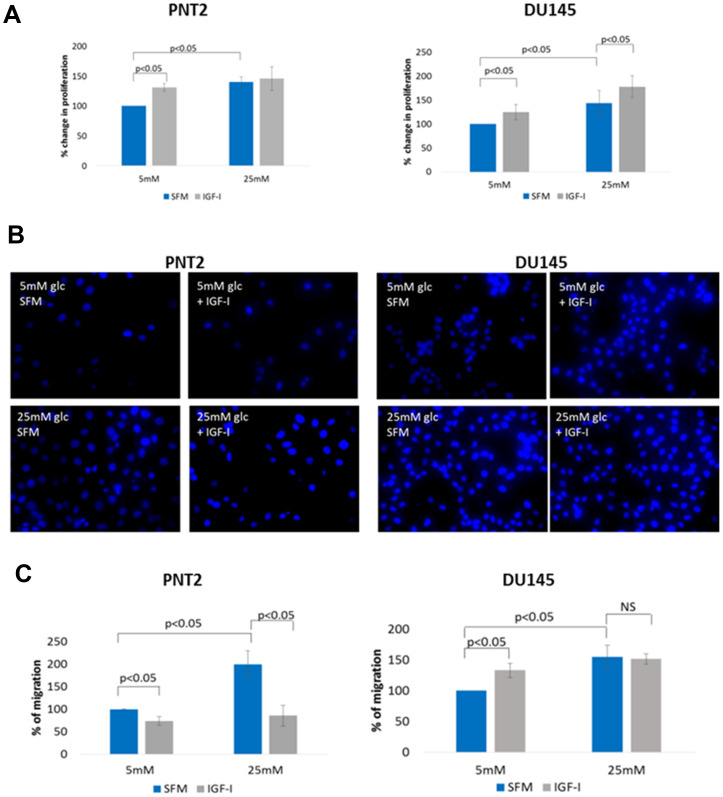
The effect of IGF on cell proliferation and migration in prostate epithelial cells in altered glucose condition. (**A**) PNT2 and DU145 cells were treated with 100 ng/ml IGF-I for 48 hours in normal (5 mM) and high (25 mM) glucose condition and changes in cell proliferation were assessed by direct count of viable cells in a haemocytometer. The plotted values represent means of percentage change relative to control. (**B**) Cell migration was measured using the transwell migration assay. PNT2 and DU145 cells were plated in the upper chamber of the inserts and treated with IGF-I (100 ng/ml) under normal (5 mM) and high (25 mM) glucose conditions. After 17 h, cells in the insert (non-migrated cells) were eliminated by scraping and migrated cells were stained with 4’,6-diamidino-2-phenylindole (DAPI) and counted under flourescence microscope. (**C**) Graph shows the percentage of cells migrated relative to control (non treated in normal glucose condition). All results shown are representative of three independent experiments. Data are represented as mean±SEM. Significant value observed as *p* < 0.05.

With PNT2 cells, addition of IGF-I in normal glucose conditions induced an increase in cell proliferation (*p* < 0.05) similarly to when the cells were grown in high glucose conditions alone (*p* < 0.05). However, addition of IGF-I in high glucose conditions was not able to induce a further increase in cell proliferation (Figure 2A).

With DU145 cells, addition of IGF-I in normal glucose conditions induced an increase in cell proliferation (*p* < 0,05) similarly to when the cells were grown in high glucose conditions alone (*p* < 0.05). In contrast to PNT2 cells, with DU145 cells, addition of IGF-I in high glucose conditions further increased cell proliferation significantly when compared to high glucose alone (*p* < 0.05)(Figure 2A).

Even though similar proliferative effects of IGF-I were observed in the PNT2 and DU145 cells, its effect on the migratory ability was different. Figure 2B and 2C show that with 5 mM glucose, IGF-I decreased migration of normal PNT2 cells but promoted migration of the DU145 prostate cancer cells: this reflected the changes observed in the EMT markers upon IGF exposure. However, exposure of PNT2 cells to high glucose alone markedly increased cell migration (*p* < 0.05) but was still inhibited in the presence of IGF-I (*p* < 0.05). Similar to PNT2 cells, exposure to high glucose alone increased DU145 cell migration (*p* < 0.05) but the addition of IGF-I under high glucose conditions could not enhance migration further.

### Effect of IGF-I on AKT and MAPK signaling in prostate epithelial cells in altered glucose conditions

**Figure 3:**
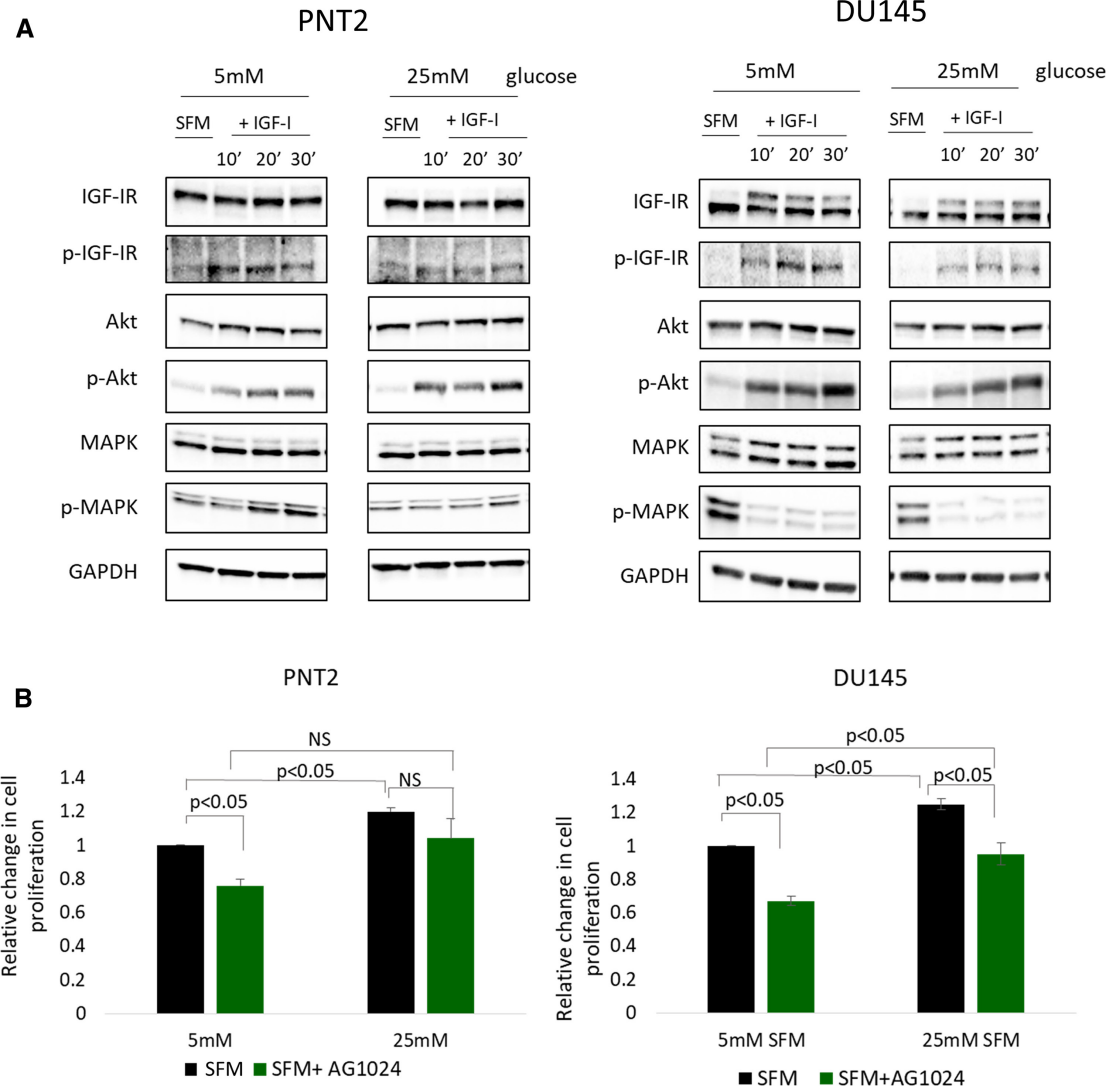
High glucose-induced EMT in prostate cells is independent of IGFI-R. (**A**) Western blot image shows the effect of IGF-I on the phosphorylation of IGF-IR, Akt and MAPK in PNT2 and DU145 cells. Cells were dosed with IGF-I 100 ng/ml for 10, 20 and 30 minutes in normal (5 mM) and high (25 mM) glucose serum free media (SFM). (**B**) Graph shows relative changes in cells proliferation in PNT2 and DU145 cells after IGFI-R receptor inhibition using tyrosine kinase inhibitor (AG1024) in different glucose conditions. Results shown are representative of at least three independent experiments. Data are represented as mean±SEM.

With PNT2 cells, addition of IGF-I in normal and high glucose conditions increased phosphorylation of the IGF-IR, AKT and MAPK time dependently. With DU145 cells, the IGF-IR and its downstream target AKT were also activated upon treatment with IGF-I, whereas IGF-I decreased the phosphorylation of MAPK. These results are consistent with the action of IGF-I in regulating EMT in both cell lines, being dependent on the IGF-IR in normal and high glucose conditions. However, exposing both PNT2 and DU145 to high glucose conditions alone [25 mM compared with 5 mM] did not activate the IGF pathway despite the promotion of EMT observed in these cells suggesting that the induction of EMT by high glucose was not regulated through activation of the IGF pathway (Figure 3A). To further verify that the effect of high glucose was independent of IGF-IR signalling in PNT2 and DU145 cells, the activation of the IGF-IR was blocked using an IGF-IR inhibitor, AG1024 and the changes in cell proliferation were examined. With PNT2 cells, inhibition of the IGF-IR was found to significantly reduce cell proliferation in normal glucose conditions by approximately 25% (*p* < 0.05). However, inhibition of the IGF-IR in high glucose conditions was not able to inhibit high glucose-induced proliferation of PNT2 cells. With DU145 cells, high glucose alone increased cell proliferation. Similarly, inhibition of the IGF-IR in normal glucose conditions inhibited proliferation of DU145 cells, as had been observed with PNT2 cells. In high glucose conditions, IGF-IR inhibition was also found to partially reduce DU145 cell proliferation (*p* < 0.05) but was still higher when compared to IGF-IR inhibition in normal glucose condition (*p* < 0.05)(Figure 3B). Taken together, these findings show that the effects of high glucose in inducing PNT2 and DU145 cell proliferation were independent of the type I IGF receptor; suggesting that an alternative pathway might be involved in mediating the effects of high glucose in promoting proliferation in prostate cells.

### Association between IGFBP-2 and FOXA1 in prostate epithelial cells

We analysed the levels of FOXA1 and IGFBP-2 in a panel of prostate cancer and normal prostate epithelial cells (Supplementary Figure 2). Although there was great heterogeneity among the cell lines, with most of the cell lines, there was a good correlation between basal FOXA1 and IGFBP-2 abundance. FOXA1 was found to be highly expressed in VCaP, LNCaP and PC3 cells. A very low level of FOXA1 was found in non-malignant PNT2 cells and malignant DU145 cells. IGFBP-2 expression was also found to be high in VCaP and LNCaP cells but very low in DU145 cells. With PC3 cells, the expression of FOXA1 and IGFBP-2 did not follow the same pattern observed in other cell lines. High FOXA1 but low IGFBP-2 levels were found in PC3 cells. Although there seemed to be no abundance of IGFBP-2 in PNT2 and PC3 cells this was likely due to the other bands corresponding to VCaP and LNCaP being excessively strong resulting in the imager not detecting the fainter bands.

**Figure 4:**
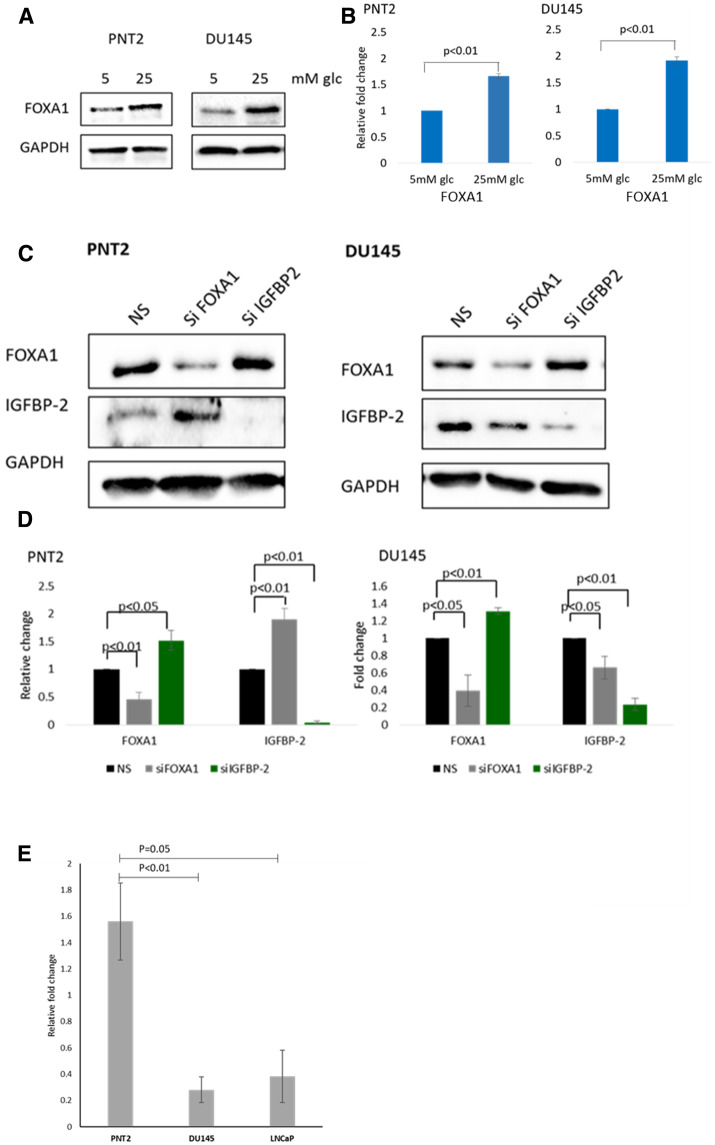
Association between IGFBP-2 and FOXA1. (**A**) Western blot image showing the effect of different glucose concentration on the level of FOXA1 in PNT2 and DU145 cells. (**B**) Optical densities of protein blots were quantitated using image J and normalised to GAPDH. (**C**) Western blot image showing the effect of stable knockdown of FOXA1 and IGFBP-2 on IGFBP-2 and FOXA1 levels in PNT2 and DU145 cells in high glucose conditions. (**D**) Optical densities of protein blots were quantitated using image J and normalised to GAPDH. The data expressed as relative changes relative to control (non-silencing). All results shown are representative of three independent experiments. Data are represented as mean ± SEM. Significant value observed as *p* < 0.05 (**E**) Results from real time PCR showing relative fold change levels of FOXA1 protein associating with the *IGFBP-2* gene associated with normal epithelial prostate cells PNT2 and two prostate cancer cell lines DU145 and LNCaP, in high glucose conditions, after performing a chromatin immunoprecipitation (ChIP) assay. Graph represents the mean of relative fold change from three separate experiments (*n* = 3).

Figure 4A and 4B show that hyperglycaemia induced a large increase in the level of FOXA1 in both PNT2 and DU145 cell lines (this was also observed with LNCaP cells, Supplementary Figure 1B). Having shown previously that IGFBP-2 is also regulated by hyperglycaemia we investigated if these two molecules might regulate EMT in prostate cells exposed to high levels of glucose. First, we investigated if there was an association between IGFBP-2 and FOXA1. We examined the effect of FOXA1 silencing on IGFBP-2 abundance in PNT2 and DU145 cells. With PNT2 cells, FOXA1 silencing induced a significant increase in IGFBP-2 (*p* < 0.01). However, with DU145 cells, a different effect was observed with a a significant reduction (*p* < 0.05) in the level of IGFBP-2 when FOXA1 was silenced when compared to a non-silencing control (Figure 4C and 4D; this was also observed with LNCaP cells, Supplementary Figure 1C). These data suggest that FOXA1 is a negative regulator of IGFBP-2 in normal but not in the cancer cells. Successful knock-down of FOXA1 is shown by western blot image and graphs of optical density measurements for FOXA1 in all cell lines. Having shown that FOXA1 regulated IGFBP-2 levels, we wanted to know whether modulating IGFBP-2 expression could in turn affect abundance of FOXA1. With PNT2 and DU145 cells, IGFBP-2 depletion resulted in increased FOXA1 abundance (*p* < 0.05 and *p* < 0.01 respectively) (Figure 4C and 4D). Using CHiP analysis, we then assessed if there was an interaction between FOXA1 and the *IGFBP-2* gene. Compared with PNT2 cells, the association was decreased by 82% (*p* < 0.01) in DU145 cells and by 76% (*p =* 0.05) in LNCaP cells (Figure 4E), indicating a strong interaction between FOXA1 and the *IGFBP-2* gene in normal prostate cells and a markedly reduced interaction between the two in cancer cells

### A role for FOXA1/IGFBP-2 in high glucose-induced EMT

**Figure 5:**
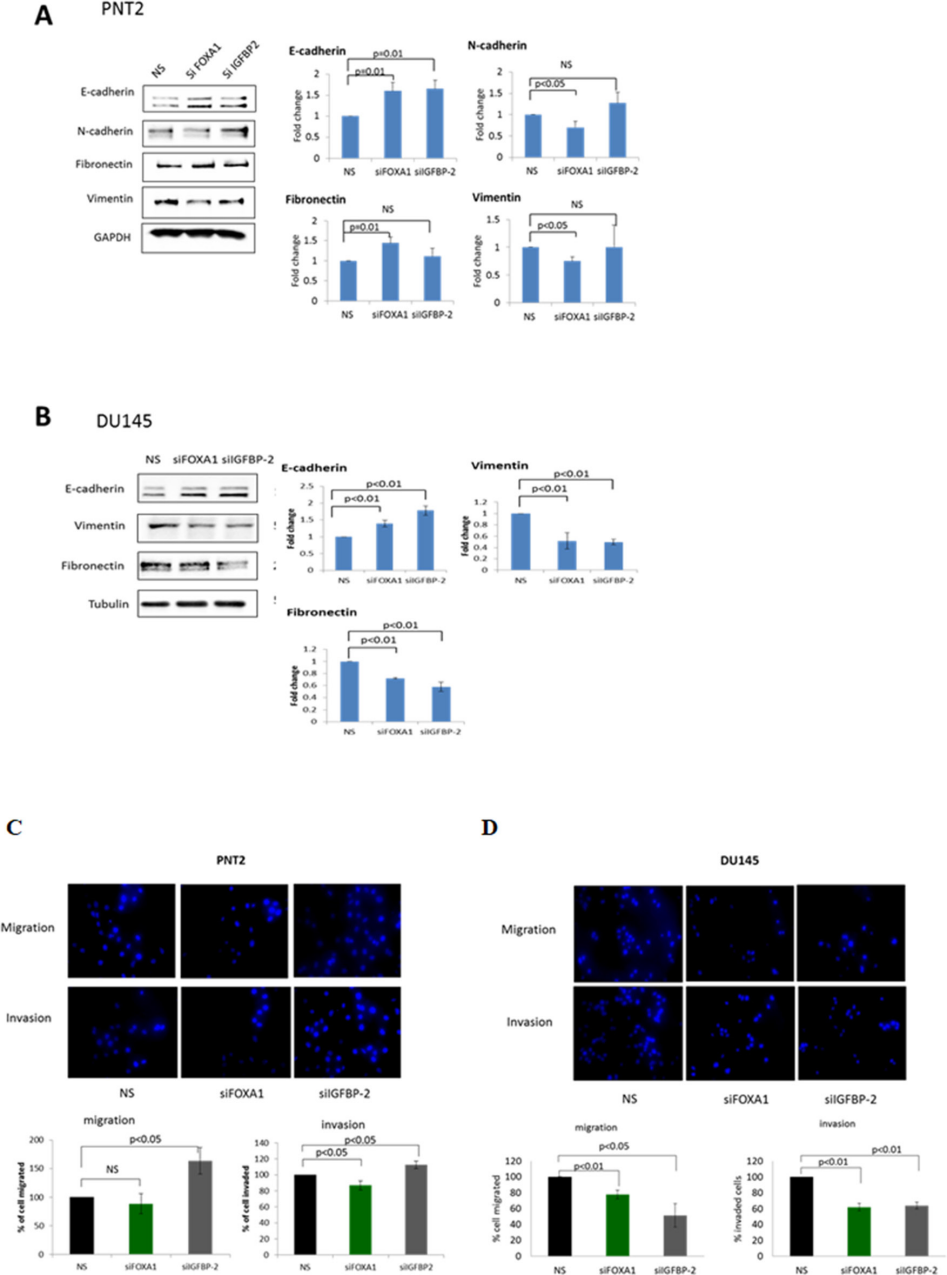
A role for FOXA1/IGFBP-2 in high glucose-induced EMT Western blots images showing the effect of stable knock down of FOXA1 and IGFBP-2 on EMT in (**A**) PNT2 and (**B**) DU145 cells. Graphs represent the western blots images and show the optical densities of the blots quantitated using image J and normalised to GAPDH/Tubulin. The data are expressed as fold changes relative to control (non-silencing). (**C**), (**D**) The effect of stable knock down of FOXA1 and IGFBP-2 on migration and invasion of PNT2 and DU145 cell using transwell migration/invasion assay. FOXA1 and IGFBP-2 were knocked down in PNT2 and DU145 cells for 72 hours, trypsinised, counted and plated in the upper chamber of the inserts. After 17 h, cells in the insert (non-migrated cells) were eliminated by scraping and migrated cells were stained with 4’,6-diamidino-2-phenylindole (DAPI) and counted under flourescence microscope. Graphs below each image show the percentage of cells migrated and invaded relative to control (non silencing). All results shown are representative of three independent experiments. Data are represented as mean±SEM. Significant value observed as *p* < 0.05

Silencing of FOXA1 in PNT2 cells promoted a more epithelial-like phenotype as demonstrated by a significant increase in the epithelial marker E-cadherin (*p =* 0.01) and a significant reduction in the mesenchymal markers N-cadherin (*p* < 0.05) and vimentin (*p* < 0.05). An unexpected increase in fibronectin was also observed when FOXA1 was silenced. Similarly, IGFBP-2 silencing in PNT2 resulted in a significant increase in E-cadherin levels (*p =* 0.01) but no significant changes were observed in mesenchymal markers (Figure 5A).

With DU145 cells, FOXA1 and IGFBP-2 silencing were both found to repress EMT as demonstrated by an up-regulation of E-cadherin by (*p =* 0.01 and *p =* 0.01) respectively when compared to a non-silencing control. Depletion of FOXA1 and IGFBP-2 levels were also found to reduce vimentin (*p* < 0.01) and fibronectin (*p* < 0.01 and *p* < 0.01) respectively (Figure 5B).

With PNT2 cells, FOXA1 silencing did not alter the migratory ability but induced a slight reduction in cell invasion (*p* < 0.05). Unexpectedly, IGFBP-2 silencing induced cell migration and invasion by approximately 60% (*p* < 0.05) and 15% (*p* < 0.05) respectively (Figure 5C). Interestingly, with DU145 cells, FOXA1 silencing induced a reduction in cell migration and invasion by 25% (*p* < 0.01) and 40% (*p* < 0.01) respectively. Similar findings were observed when IGFBP-2 was silenced; a reduction in the migratory and invasive potential of DU145 by 50% (*p* < 0.05) and 40% (*p* < 0.01) respectively were found (Figure 5D).

### Positive association between FOXA1 and IGFBP-2 level in prostate cancer tissue

**Figure 6:**
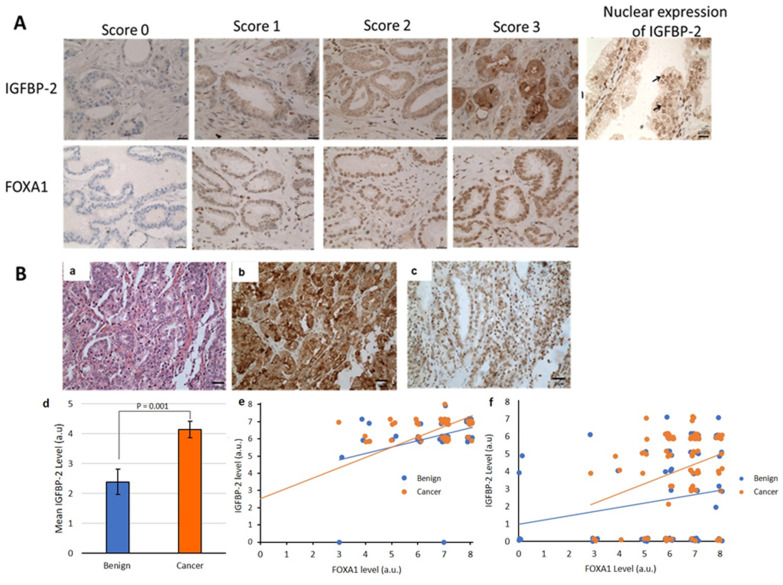
(**A**) IGFBP-2 and FOXA1 expression in prostate cancer tissue. Tissue demonstrating negative staining (0 score), weak staining (1 score), moderate staining (3 score), strong staining (3 score) (×20 magnification) and positive IGFBP-2 nuclear expression in prostate epithelial cells (see →) (×40 magnification). (**B**) Association between FOXA1 and IGFBP-2 levels in prostate cancer tissue. (a) Hematoxylin and Eosin staining (b) strong IGFBP-2 and (c) FOXA1 staining in the same prostate cancer tissue (×20 magnification). (d) bar chart showing the significant increase in IGFBP-2 expression in cancer compared to benign tissue in the PrEvENT cohort and scatter plots showing the significant positive association between FOXA1 and IGFBP-2 proteins level in prostate cancer tissue in the Southmead (e) and PrEvENT (f) cohorts.

As shown in Figure 6, IGFBP-2 was predominantly confined to the cytoplasm with weak stromal staining. Interestingly, nuclear localisation of IGFBP-2 was also observed in both adjacent benign and prostate cancer cases (Figure 6A). IGFBP-2 was observed in both adjacent benign tissue as well as in the cancer region and showed no significant difference between them in the smaller cohort (*p =* 0.274), however in the larger PrEvENT cohort, IGFBP-2 expression was significantly increased in cancer compared to benign tissue (*p =* 0.001). Correlation analysis between IGFBP-2 and clinicopathological parameters of prostate cancer showed that IGFBP-2 was not significantly associated with age (*p =* 0.834), pre-operative PSA level (*p =* 0.051), Gleason score (*p =* 0.192), pathologic stage (*p =* 0.813) or lymph node involvement (*p =* 0.850) in the Southmead cohort (Table 1). Similarly, in the PrEvENT cohort there was no significant correlation between IGFBP-2 and age (*p =* 0.432), pre-operative PSA (*p =* 0.354) or pathologic stage (*p =* 0.922), however there was a significant association with Gleason Score (*p =* 0.022). We were not able to statistically assess the association with lymph node invasion in this cohort due to this information being unavailable for many of the patients.

**Table 1:** Clinical and pathological features of the Southmead and PrEvENT prostate tissue cohorts and their association with FOXA1 and IGFBP-2 expression

Variables		Southmead Cohort			PrEvENT Cohort		
		Median (range) or n (%)	P for c2		Median (range) or n (%)		P for c2	
			FOXA1	IGFBP-2		FOXA1	IGFBP-2
***N***		47			95		
**Age at Surgery**		64 (46-76)	0.582	0.834	65 (45-74)	0.711	0.432
	≤**64**	24 (51.1%)			47 (49.5%)		
	**>64**	23 (48.9%)			48 (50.5%)		
**Pre-operative PSA (ng/ml)**		11.7 (1.2-74)	0.288	0.051	8.6 (2.2-70)	0.209	0.354
	**<10**	21 (44.7%)			55 (57.9%)		
	**10-20**	15 (31.9%)			28 (29.5%)		
	**>20**	11 (23.4%)			6 (6.3%)		
	**Unknown**	0 (0%)			6 (6.3%)		
**Gleason Grade**			0.645	0.192		0.27	0.022
	**<7**	14 (29.7%)			11 (11.6%)		
	**7**	21 (44.7%)			76 (80%)		
	**>7**	12 (25.5%)			6 (6.3%)		
	**Unknown**	0 (0%)			2 (2.1%)		
**Pathologic Stage**			0.544	0.813		0.279	0.922
	**pT2**	14 (29.7%)			28 (29.5%)		
	**pT3a**	21 (44.7%)			44 (46.3%)		
	**pT3b**	12 (25.5%)			12 (12.6%)		
	**pT4**	0 (0%)			1 (1.1%)		
	**Unknown**	0 (0%)			10 (10.5%)		
**Lymph node invasion**			0.787	0.850	Insufficient information to perform statistical analysis
	**No**	26 (55.3%)					
	**Yes**	17 (36.2%)					
	**Unknown**	4 (8.5%)					

*P* values indicate any changes in expression levels with clinicopathological features across the range of each cohort.

FOXA1 was found to be predominantly confined to the nucleus of epithelial prostate cancer cells as well as in adjacent benign tissue (Figure 6A). There was no significant difference observed in the FOXA1 staining between these tissues in either cohort (*p =* 0.665, *p =* 0.151). In both cohorts, the expression of FOXA1 was also not significantly associated with age (*p =* 0.582, *p =* 0.711), pre-operative PSA levels (*p =* 0.288, *p =* 0.209), Gleason score (*p =* 0.645, *p =* 0.270), pathologic stage (*p =* 0.544, *p =* 0.279) or lymph node involvement (*p =* 0.787, *p =* 0.114)(Table 1). Interestingly, in the smaller tissue cohort we found a positive correlation between FOXA1 and IGFBP-2 levels (r = 0.319, *p =* 0.029) in prostate cancer tissue. Although, in benign tissue, there was a trend for positive correlation between these proteins, this was not statistically significant (r = 0.259, *p =* 0.079) (Figure 6B). In the larger PrEvENT cohort, the association was significant in both benign tissue (r = 0.249, *p =* 0.014) and cancer tissue (r = 0.219, *p =* 0.033).

This finding supports the evidence from the *in vitro* study that showed an association between FOXA1 and IGFBP-2 in prostate epithelial cells.

## DISCUSSION

The bioactivity of IGF-I is intricately integrated with nutritional status and energy balance; and alterations in metabolism associated with adoption of a western lifestyle have been postulated to contribute to the increased risk of developing prostate cancer [27]. Increased expression of IGF-I has been associated with poor prognosis and more aggressive cancers that exhibit increased metabolism and increased glucose uptake. The activation of the EMT program contributes to the progression of metastatic prostate cancer, which is the principle cause of death in most of prostate cancer patients. In this study we investigated the role of IGF-I in promoting prostate cancer progression through activation of the EMT program. With normal PNT2 cells, under euglycaemic conditions, EMT was inhibited by IGF-I, whereas opposite effects were observed in the cancer cells, which suggest that with normal epithelial prostate cells, IGF-I prevented a malignant phenotype by maintaining the normal characteristics of differentiated cells whereas in cancer cells, IGF-I promoted a more mesenchymal phenotype that could potentially promote a more aggressive phenotype.

The ability of IGF-I to induce EMT in prostate cancer cells was also consistent with studies conducted in brain [28] and breast [29] cancer cell lines. Similar observations were made by Graham *et al* (2015) where IGF-I induced EMT in ARCaP prostate cancer cells through up-regulation of Zinc finger E-box-binding homeobox (ZEB1), a transcription factor that regulated EMT activation [30].

IGF-I induced cell proliferation in both cancer and non-cancerous cells confirming its mitogenic role in regulating cell growth and differentiation in wide variety of cells as reported in the literature [31, 32]. Even though similar proliferative effects of IGF-I were observed in cancer and non-cancerous cells, its effect on the migratory ability was different. IGF-I decreased migration of normal PNT2 cells but promoted migration of the DU145 prostate cancer cells: this reflected the changes observed in the EMT markers upon IGF exposure. Increases in E-cadherin by IGF-I that was observed with PNT2 cells, tightens the cell-cell junctions thus limiting the ability of cells to move and migrate. On the other hand, with DU145 cells, IGF-I increased the cells’ potential for metastasis as evidenced by increases in cell migration.

Epidemiology studies have shown a positive association between metabolic syndrome such as obesity and diabetes with cancer development and mortality. Increases in IGF-I levels appear to be one of the factors linking these different diseases [33]. Hyperglycaemia, a hallmark of diabetes, has been shown to promote cancer progression [7]. We have also shown previously that hyperglycaemia induced chemoresistance in prostate cancer cells [18]. In this study, we revealed for the first time that exposure to high concentrations of glucose (25 mM) alone induced EMT in both cancer and non-cancerous prostate epithelial cells compared to normal glucose (5 mM) conditions. The transition from epithelial to mesenchymal characteristics in these cell lines was also correlated with increases in proliferation and migratory potential in both cell lines. More interestingly, with PNT2 cells, despite the ability of hyperglycaemia alone to induce EMT, the effects were still reversed by addition of IGF-I: IGF-I was able to promote an epithelial phenotype in PNT2 cells in both normal and high levels of glucose. However, with DU145 cells, addition of IGF-I in high glucose conditions was not able to augment the effect of high glucose alone on EMT. These data suggested that different concentrations of glucose do not influence the action of IGF-I and that glucose alone can independently promote tumour aggressiveness and metastasis.

The involvement of hyperglycaemia in prostate cancer cell EMT, progression and metastasis may underpin why prostate cancer patients that present with hyperglycaemia have a worse prognosis. High intake of total energy has been associated with increased risk of fatal prostate cancer [34]. Dietary restriction and lifestyle changes are suggested as an intervention for suppresion of cancer growth. A study of calorie restriction performed in mice showed a reduction in circulating IGF-I and insulin levels and deactivation of the PI3K/AKT pathway which resulted in prostate tumour growth inhibition [35]. In addition, calorie restriction combined with IGF-1R blockade resulted in growth inhibition in prostate cancer xenografts [36]. Taken together, this evidence suggests that limiting energy consumption may reduce the risk of prostate cancer progression by improving the metabolic profile.

β-catenin plays an important role in both maintaining epithelial integrity and as a co-activator of Wnt-mediated gene transcription. The activation of Wnt/ β-Catenin signalling has been shown to promote EMT in prostate cancer. This study revealed that IGF-I promoted destabilization of β-catenin by inducing its phosphorylation at Ser33/37/T41 in both PNT2 and DU145 cells and that this effect was not affected by levels of glucose. It is also interesting to note that despite this observation (destabilization of β-catenin), IGF-I also induced β-catenin nuclear translocation, which is indicative of Wnt/β-catenin transcriptional activity. Likewise, increased hyperglycaemic conditions alone had the same effect on β-catenin phosphorylation and nuclear translocation in DU145 in contrast to the PNT2 cells although in both cell lines hyperglycaemia yielded a similar effect on EMT.

IGF-I exerts its biological effects through binding to its receptor, predominantly the IGF-IR, activating downstream signalling cascades. With PNT2 cells, both IGF-IR/PI3K/AKT and IGF-IR/RAS/MAPK pathways were found to be equally activated by IGF-I whereas with DU145 cells, IGF-IR/PI3K/AKT appeared to be the dominant signalling pathway in mediating IGF-I-induced EMT in DU145 cells. In contrast to IGF-I, hyperglycaemia did not induce activation of the IGF-IR or components of the IGF-IR signalling pathway (p-AKT or p-MAPK) in either cell model although all the features of EMT were enhanced by hyperglycaemia in a similar way to IGF-I in euglycaemic conditions. This suggests that hyperglycaemia-induced EMT in PNT2 and DU145 cells was independent of IGF signalling, that was confirmed using AG1024 a tyrosine kinase inhibitor to block IGF-IR activation. In euglycaemic conditions, blocking the IGF-IR reduced cell growth as expected, however in hyperglycaemic conditions, the hyperglycaemia-induced effect of increasing cell proliferation was unaffected. This shows that a different mechanism in which hyperglycaemia activates the EMT program was involved: one of which could be through the regulation by IGFBP-2. There is accumulating evidence that IGFBP-2 may have an important role in prostate cancer progression [37]. We have shown previously that IGFBP-2 promotes prostate cancer growth in both an IGF-I-dependent and independent manner [17] and that hyperglycaemia induced up-regulation of IGFBP-2 in prostate cancer cells which resulted in resistance to chemotherapy [18].

Apart from IGFBP-2, an increase in the level of FOXA1 by hyperglycaemia in prostate cell lines was also observed in this study. In light of these findings, we investigated if there was interplay between IGFBP-2 and FOXA1. Our *in vivo* study indicated that a positive correlation existed between levels of IGFBP-2 and FOXA1 in benign and cancerous prostate tissue samples. With the CHiP analysis using cell lines, we found that FOXA1 strongly interacted with the *IGFBP-2* gene in normal prostate epithelial. The siRNA data showed that silencing FOXA1 in normal prostate cells resulted in a large increase in IGFBP-2. Collectively, these data may suggest that in normal prostate epithelial cells, FOXA1 binds to the *IGFBP-2* gene to negatively regulate IGFBP-2 levels.

With the cancer cells, CHiP analysis indicated that the level of FOXA1 associating with the *IGFBP2* gene was minimal in comparison to the normal cells. siRNA also indicated that the relationship between FOXA1 and IGFBP-2 in cancer cells was different, as although silencing FOXA1 reduced levels of IGFBP-2, the ratio of FOXA1 to IGFBP-2 appeared to remain the same.

It is well established that IGFBP-2 is upregulated in many cancers including the prostate and this was observed in our own tissue cohort. Our data may suggest that FOXA1 keeps IGFBP-2 in check in normal cells, but this negative regulation is lost in the cancer cells.

Next, we investigated the ability of FOXA1 and IGFBP-2 to regulate EMT in prostate epithelial cells: FOXA1 silencing in PNT2 and DU145 cells resulted in a more epithelial-like phenotype manifested by an increase in the epithelial marker, E-cadherin, and a reduction in mesenchymal markers such as vimentin and fibronectin. Cell migration and invasion were also inhibited following FOXA1 silencing. This would be consistent with a previous report that revealed higher expression of FOXA1 in prostate cancer than in normal prostate tissue that was associated with advanced stages of cancer [24]. Moreover, overexpression of FOXA1 was also found in metastatic and castration-resistance prostate cancer that correlated with higher pT stage and Gleason score [38]. FOXA1 silencing in prostate cells was found to inhibit cell growth and induced G0/G1 arrest [24] as well as induce a reduction in cell migration [38]. However, despite all the evidence on the oncogenic role of FOXA1 in prostate cancer as discussed above, there are also reports that reveal its role as a suppressor of EMT. Jin *et al* showed that high expression of FOXA1 suppressed EMT, migration and invasion in prostate cancer by directly inhibiting SLUG gene expression [21]. Similar findings were also reported in pancreatic ductal adenocarcinoma [20]. Most recently, a study by Huang *et al* revealed that TRPS1-induced FOXA1 transcriptional activity inhibited EMT and metastasis in breast cancer cells [39]. Similarly, to FOXA1, IGFBP-2 silencing in DU145 resulted in total reversal of EMT, inhibition of migration and invasion. Meanwhile with PNT2 cells, IGFBP-2 silencing resulted in partial repression of EMT as demonstrated by up-regulation in the epithelial marker E-cadherin but no observed changes in mesenchymal markers. Surprisingly, the migratory and invasive potential of PNT2 cells were increased with a reduction in IGFBP-2 levels.

These data suggest that FOXA1 and IGFBP-2 each contribute to the process of glucose-induced EMT and that the level of binding of FOXA1 with the *IGFBP* gene plays a role. In summary, IGF-I and hyperglycaemia-induced FOXA1/IGFBP-2 play important roles in promoting prostate cancer cell progression. These data may underpin why men with prostate cancer, who present with hyperglycaemia, have a worse prognosis and may lead to new potential therapeutic strategies.

## MATERIALS AND METHODS

Human, recombinant IGF-I was purchased from Gropep (Adelaide, Australia) and an IGF-IR inhibitor, AG1024, was obtained from Sigma-Aldrich, Dorset, UK.

### Cell culture

A normal epithelial prostate cell line, PNT2 was purchased from ECACC (London, UK) and DU145 and LNCaP prostate cancer cells were purchased from ATCC (Teddington, Middlesex, UK). All cell lines were cultured as described previously [18]. These cells have been authenticated by short tandem repeat (STR) analysis and had been confirmed as mycoplasma-negative in our routine quality control.

### Cell proliferation assay

Trypan Blue dye exclusion assay was performed to assess cell proliferation as described previously [40].

### Transwell migration and invasion assay

These were performed as described previously [41]. To assess the migratory potential, cells were seeded into the upper chamber of a cell permeable membrane of 8 mm pore trans-well inserts and to create an invasion assay, the cell permeable membrane was coated with collagen (1%), to mimic the extracellular matrix that cells encounter during the invasion process. 1% solution v/v of collagen type I was prepared by diluting stock solution with PBS. 200ml of working solution was added to the upper chamber of the trans-well inserts. The inserts were dried overnight prior to seeding the cells.

### siRNA and transfections

IGFBP-2 siRNA was used as described previously [18]. ON-TARGET*plus* SMARTpool FOXA1 siRNAs were bought from Dharmacon Thermo Scientific (Lafayette, CO, USA). The transfection kit was purchased from Synvolux Therapeutics (Leiden, The Netherlands) and transfection was conducted following the manufacturer’s instructions.

### Western immunoblotting

Western blot analysis was performed as described previously [40]. Briefly, 30 μg of protein were run on 10% SDS-PAGE, transferred to nitrocellulose membrane (BioRad, Hertfordshire, UK) and immunoblotted with the following antibodies: E-cadherin (1:1000, Cell Signaling Hertfordshire, UK), vimentin (1:500, BD Biosciences Oxford, UK), fibronectin (1:500, BD Biosciences Oxford, UK), FOXA1(1:100, Abcam), IGFBP-2(1:1000, Santa Cruz Heidelberg, Germany), MAPK (Erk1/2)(1:1000, Cell Signaling Hertfordshire, UK), AKT (1:1000, Cell Signaling Hertfordshire, UK), p-AKT (S473)(1:1000, Cell Signaling Hertfordshire, UK), p-IGF1-R/IR (1:1000, Cell Signalling), p- MAPK (Erk1/2; Thr202/Tyr204)(1:1000, Cell Signaling Hertfordshire, UK), GAPDH (1:5000, Merck Millipore Hertfordshire, UK) and tubulin (1:5000, Merck Millipore Hertfordshire, UK), following the manufacturer’s instructions. After incubation with specific secondary antibodies conjugated to peroxidase (Sigma-Aldrich, Dorset, UK.), proteins were visualised by Clarity ECL substrate (BioRad, Hertfordshire, UK) using BioRad Chemidoc XRS + system and analysed using Image lab software (BioRad, Hertfordshire, UK).

### Chromatin immunoprecipitation (ChIP) assay

A ChIP assay was performed in order to assess the interaction between FOXA1 and the *Igfbp2* promoter. The Pierce Agarose ChIP Kit (Thermo Scientific) was used and was carried out as per the manufacturer’s instructions. Briefly, cells were cultured under conditions of high glucose and 2 × 10^6^ cells per ChIP reaction were cross-linked with formaldehyde and digested by Micrococcal Nuclease Digestion. DNA fragments bound to FOXA1 were immunoprecipitated using a ChIP-grade antibody (5μg per reaction, Thermo Fisher). DNA was recovered and cleaned then qPCR performed as we have described previously [18] to determine the quantitity of *IGFBP-2*-bound to FOXA1.

### Tissue samples

We obtained two separate cohorts of human prostate tissue samples, the clinical and pathological data of which are summarised in Table 1. The first cohort of 47 samples was collected from patients with prostate cancer that underwent prostatectomy from years 2008-2015 The second cohort of 95 samples was collected for the Prostate: Evidence for Exercise and Nutrition (PrEvENT). Trial samples for each study were collected at Southmead Hospital, Bristol, with full ethical approval (15/WM/0449 and 14/SW/0056 respectively). For these studies men were consented to allow prostate tissue samples to be obtained from the prostate that was removed at the time of surgery [42]. All samples collected, were used and stored in accordance with the Human Tissue Act 2004 and the study was performed in accordance with the Declaration of Helsinki. Prostate tissue sections that were used were formalin-fixed and the tissue was surplus to diagnostic requirements. It was paraffin embedded before being cut to 4μm thickness and collected on adhesive slides. Patient’s clinical and pathological data including age at surgery, pre-surgery PSA level, Gleason and histological grade and lymph node invasion were extracted and analysed anonymously from medical records for the purpose of this study.

### Immunohistochemistry

All reagents were purchased from Ventana Medical Systems unless stated otherwise. The tissues were stained using a fully automated Ventana BenchMark Ultra Immunostainer system (Ventana Medical Systems) following the manufacturer’s protocol. Briefly, the cut paraffin sections were dried at 60° C for 1 hour on a microslide prior to immunostaining. Each individual slide was installed onto the slide carousel and the program was run to completion. The ventana staining procedure includes deparaffinisation and pretreatment with cell conditioner. Upon cell conditioning, a primary antibody against IGFBP-2 or FOXA1 was manually added onto each slide and incubated for 2 hours. The slides were then counterstained with Hematoxylin for 8 minutes and with bluing reagent for another 4 minutes. The slides were then dehydrated and mounted in distyrene plasticizer and xylenemountant (DPX)(SIGMA). An Allred scoring system was used to assess IGFBP-2 and FOXA1 staining as we described previously [43].

### Statistical analyses

Data are represented as the mean ± SEM from three independent experiments each repeated in triplicate unless otherwise stated. Statistical analysis was performed using IBM SPSS statistics 23(IBM Corporation, version 23.0.0.2). Independent samples t-test was used to compare means of two groups. One-way Analysis of variance (ANOVA) was used for multiple comparisons followed by least significant difference (LSD) post hoc test. A significant difference was considered present at *p* < 0.05. CHIP experiment data were analysed using a fold enrichment method (alternatively called “signal over background”) where dCt values (delta cycle threshold) were calculated by subtracting Ct for negative control (no antibody) from Ct values for individual samples. Next a fold enrichment method was calculated using formula 2^-(dCt) for each individual sample. The ChIP assay was performed three times and statistical differences between the means of these fold changes were calculated using one-way Anova with Least Significant Difference post-hoc test at significance level equal to 0.05. Differences in FOXA1 and IGFBP-2 staining between benign and prostate cancer tissue were calculated using Mann-Whitney rank –sum test. The Pearson χ^2^ chi square test or Fisher’s exact test was used to test the differences between FOXA1/ IGFBP-2 abundance and clinicopathological parameters. The Spearman’s rank correlation coefficient was used to assess the correlation between IGFBP-2 and FOXA1 levels.

## SUPPLEMENTARY MATERIALS


