# Correction: IGF-1 and hyperglycaemia-induced FOXA1 and IGFBP-2 affect epithelial to mesenchymal transition in prostate epithelial cells

**DOI:** 10.18632/oncotarget.28344

**Published:** 2023-01-26

**Authors:** Rehanna Mansor, Jeff Holly, Rachel Barker, Kalina Biernacka, Hanna Zielinska, Anthony Koupparis, Edward Rowe, Jon Oxley, Alex Sewell, Richard M. Martin, Athene Lane, Lucy Hackshaw-McGeagh, Claire Perks

**Affiliations:** ^1^IGFs and Metabolic Endocrinology Group, Bristol Medical School, Translational Health Sciences, University of Bristol, Southmead Hospital, Bristol, UK; ^2^Faculty of Medicine, Royal College of Medicine Perak, Universiti Kuala Lumpur, Ipoh, MY; ^3^Department of Urology, Bristol Urological Institute, Southmead Hospital, Bristol, UK; ^4^Department of Cellular Pathology, North Bristol NHS Trust, Southmead Hospital, Bristol, UK; ^5^NIHR Biomedical Research Centre, Level 3, University Hospitals Bristol Education Centre, Bristol, UK; ^6^Population Health Sciences, University of Bristol, Bristol, UK

**This article has been corrected:** In Figure 1, the legend has been amended to show that the β-catenin blots in panel A were re-used in panels D and F as well. The corrected Figure 1 legend is shown below. The authors declare that these corrections do not change the results or conclusions of this paper.

Original article: Oncotarget. 2020; 11:2543–2559. 2543-2559. https://doi.org/10.18632/oncotarget.27650

**Figure 1:**
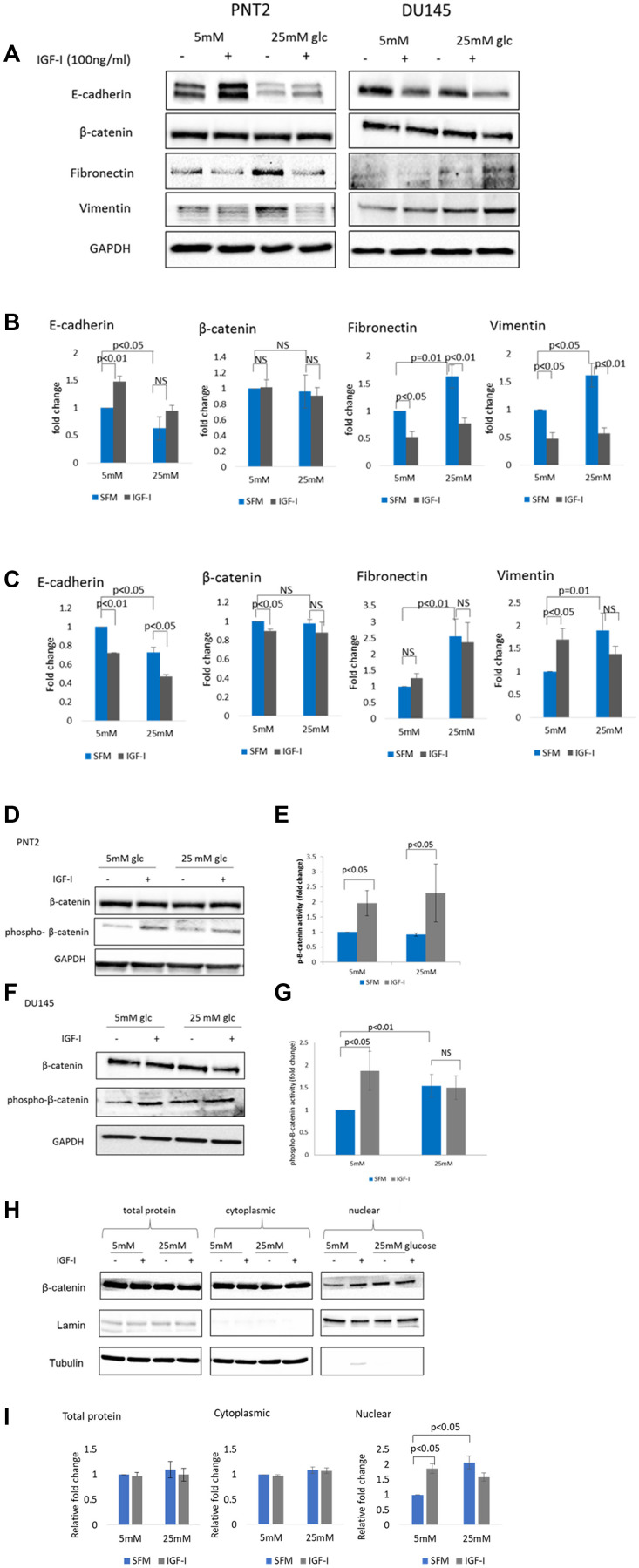
The effect of IGF-I on EMT markers in prostate epithelial cells in altered glucose condition. (**A**) Western blot image shows the effect of IGF-I and high glucose on mesenchymal markers in PNT2 and DU145 cells. Cells were dosed with IGF-I 100 ng/ml for 48 hours in normal (5 mM) and high (25 mM) glucose serum free media. Equal amounts of extracted proteins were separated by SDS-PAGE, blotted to a nitrocellulose membrane and probed with primary antibodies against E-cadherin, β-catenin, fibronectin, vimentin and GAPDH. GAPDH was used as a loading control. Optical densities of protein blots for (**B**) PNT2 and (**C**) DU145 were quantitated using image J and normalised to GAPDH. Western blots showing regulation of p-β-catenin in (**D**) PNT2 and (**F**) DU145 cells when treated with 100 ng/ml IGF-I in normal (5 mM) and high (25 mM) glucose serum free media. The β-catenin blots in (D) and (F) were reused from β-catenin blots in (A). Optical densities of protein blots for (**E**) PNT2 and (**G**) DU145 were quantitated using image J and normalised to GAPDH. Ratio of normalised total β-catenin: p- β-catenin were measured and used as an indicator of β-catenin activity. The data expressed as fold changes relative to control represent mean+/− SE of triplicate experiments. (**H**) Western blot showing cytosolic and nuclear fractions of protein separated form whole cells lysate (total protein) from DU145 cells treated or untreated with 100 ng/ml IGF-I for 48 hours in normal (5 mM) and high (25 mM) glucose serum free media. Lamin A/C and tubulin act as nuclear and cytoplasmic loading controls respectively. Results shown are representative of three independent experiments. Data are represented as mean ± SEM.

