# Supplementary material for: IGF-1 and hyperglycaemia-induced FOXA1 and IGFBP-2 affect epithelial to mesenchymal transition in prostate epithelial cells
Source: Oncotarget. 2020 Jun 30;11(26):2543–59. doi: 10.18632/oncotarget.27650 (PMC7335671; doi:10.18632/oncotarget.27650)
Supplement: Supplementary file 1 [file oncotarget-11-2543-s001.pdf]

IGF-1 and hyperglycaemia-induced FOXA1 and IGFBP-2 affect epithelial to mesenchymal transition in prostate epithelial cells

SUPPLEMENTARY MATERIALS

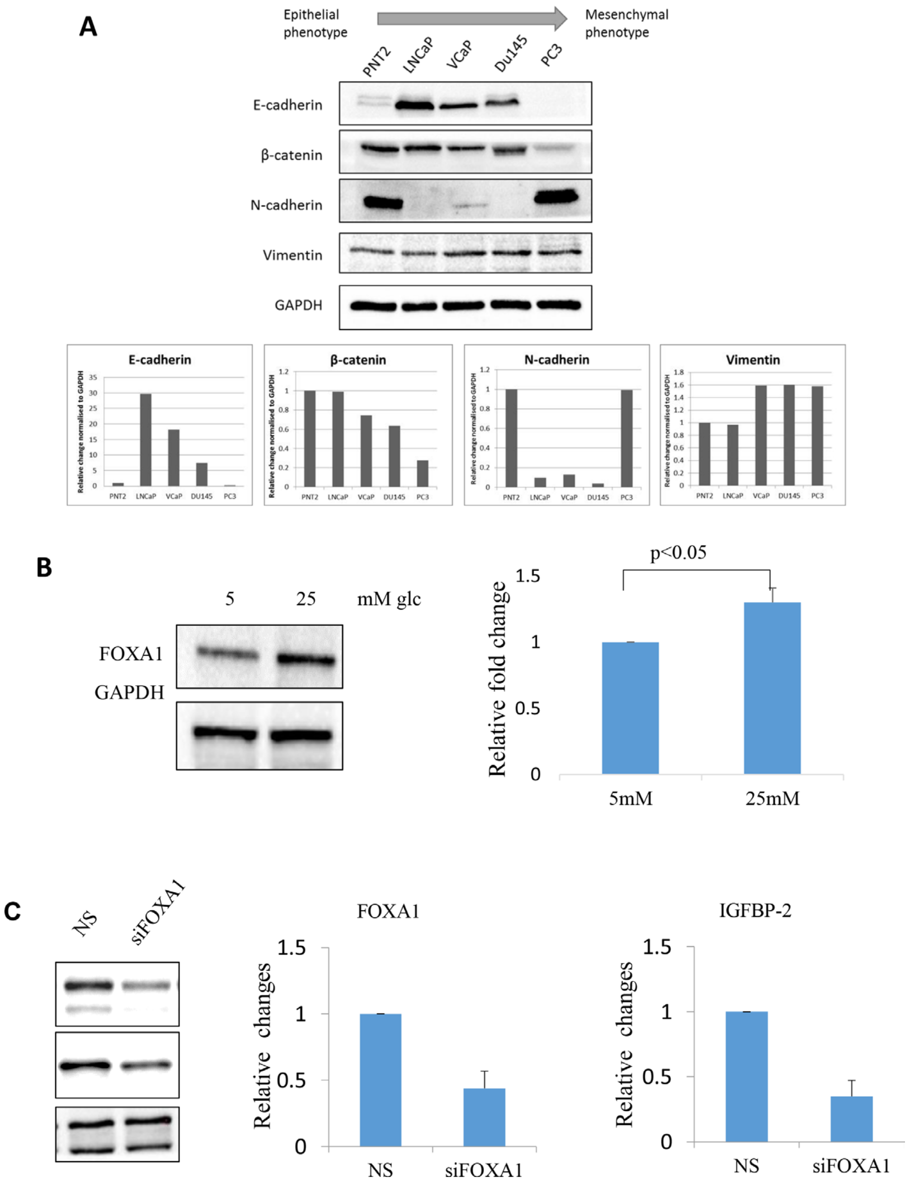

Supplementary Figure 1: (**A**) The differential levels of EMT markers in normal epithelial prostate cells and prostate cancer cell lines. Western blot showing E-cadherin, β-catenin, N-cadherin and vimentin protein levels in a panel of prostate cell lines. Prostate cell lines: PNT2, LNCaP, VCaP, DU145 and PC-3 cell lines were extracted as whole cell lysates after the cells were 80% confluence in the flask in 25 mM glucose-containing GM. Western blotting was performed to assess the abundance of the total amount of E-cadherin, β-catenin, N-cadherin and vimentin. GAPDH acted as a loading control. Quantified fold changes relative to PNT2 cells are shown in the graphs below. (**B**) Western blot image showing the effect of different glucose concentration on the level of FOXA1 in LNCAP cells. Optical densities of protein blots were quantitated using image J and normalised to GAPDH. Data are represented as mean ± SEM. (**C**) The effect of FOXA1 silencing on the level of IGFBP-2 in LNCaP cells. Cells were transfected with 50 nM FOXA1 siRNA for 72 hours using Saint Red transfection reagent. Non-silencing siRNA was used as a negative control (for knock down experiment). FOXA1 and IGFBP-2 abundance were assessed by western blot. Graphs shows efficient FOXA1 knock down and the intensity of IGFBP-2 normalized to lamin and expressed as relative fold change. (NS: Non silencing siRNA) (*n* = 2).

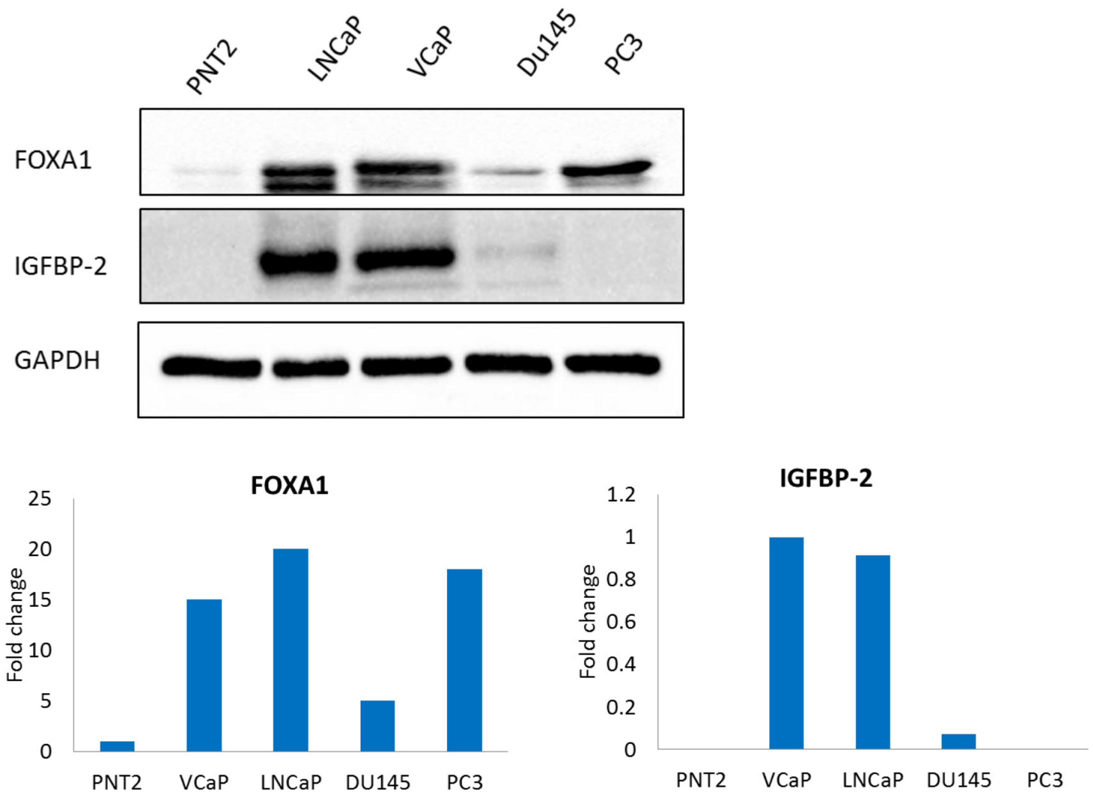

Supplementary Figure 2: The differential abundance of FOXA1 and IGFBP-2 in prostate epithelial cells. Western blot showing FOXA1 and IGFBP-2 protein levels in a panel of prostate cell lines. Prostate cell line: PNT2, LNCaP, VCaP, DU145 and PC-3 cell lines were extracted as whole cell lysates after the cells were 80% confluence in the flask in 25 mM glucose GM. Western blotting was performed to assess the abundance of the total amount of FOXA1 and IGFBP-2. GAPDH acted as a loading control. Quantified fold changes relative to PNT2 cells (for FOXA1 level) and VCaP cells (for IGFBP-2 level) are shown in the graph below the blots. (*n* = 1)
